# Six-gene signature for predicting survival in patients with head and neck squamous cell carcinoma

**DOI:** 10.18632/aging.102655

**Published:** 2020-01-12

**Authors:** Juncheng Wang, Xun Chen, Yuxi Tian, Gangcai Zhu, Yuexiang Qin, Xuan Chen, Leiming Pi, Ming Wei, Guancheng Liu, Zhexuan Li, Changhan Chen, Yunxia Lv, Gengming Cai

**Affiliations:** 1 Department of Otolaryngology, Head and Neck Surgery, Xiangya Hospital, Central South University, Changsha 410008, People’s Republic of China; 2 Department of Oral and Maxillofacial Surgery, First Affiliated Hospital of Quanzhou, Fujian Medical University, Quanzhou 362000, People’s Republic of China; 3 Department of Oncology, Xiangya Hospital, Central South University, Changsha, 410008 People’s Republic of China; 4 Department of Otolaryngology, Head and Neck Surgery, The Second Xiangya Hospital, Central South University, Changsha 410011, People’s Republic of China; 5 Department of Health Management, The Third Xiangya Hospital, Central South University, Changsha 410011, People’s Republic of China; 6 Department of Stomatology, Changzheng Hospital, Second Military Medcial University, Shanghai 200003, People’s Republic of China; 7 Department of Otolaryngology, Head and Neck Surgery, HeYuan People's Hospital, Jinan University, He Yuan 517000, People’s Republic of China; 8 Department of Otolaryngology, Head and Neck Surgery, Affiliated Hospital of Guilin University, Guilin 541000, People’s Republic of China; 9 Department of Thyroid Surgery, The Second Affiliated Hospital of Nanchang University, Nanchang 330006, People’s Republic of China; 10 Department of Otolaryngology, Head and Neck Surgery, First Affiliated Hospital of Quanzhou, Fujian Medical University, Quanzhou 362000, People’s Republic of China

**Keywords:** bioinformatics, CNV, prognostic markers, TCGA

## Abstract

The prognosis of head and neck squamous cell carcinoma (HNSCC) patients remains poor. High-throughput sequencing data have laid a solid foundation for identifying genes related to cancer prognosis, but a gene marker is needed to predict clinical outcomes in HNSCC. In our study, we downloaded RNA Seq, single nucleotide polymorphism, copy number variation, and clinical follow-up data from TCGA. The samples were randomly divided into training and test. In the training set, we screened genes and used random forests for feature selection. Gene-related prognostic models were established and validated in a test set and GEO verification set. Six genes (*PEX11A, NLRP2, SERPINE1, UPK, CTTN, D2HGDH*) were ultimately obtained through random forest feature selection. Cox regression analysis confirmed the 6-gene signature is an independent prognostic factor in HNSCC patients. This signature effectively stratified samples in the training, test, and external verification sets (P < 0.01). The 5-year survival AUC in the training and verification sets was greater than 0.74. Thus, we have constructed a 6-gene signature as a new prognostic marker for predicting survival of HNSCC patients.

## INTRODUCTION

Head and neck cancer is a highly heterogeneous malignant tumor, which can originate from various anatomical sites in the upper airway and digestive tract, including the mouth, larynx and pharynx. Most cases of head and neck cancer are squamous cell carcinoma (HNSCC), which accounts for about 4% of all new cancer diagnoses in the United States. Worldwide, there are about 600,000 new head and neck cancer patients each year [1], and the 5-year survival rate is only 40-50% [2]. The main reason for this high mortality is the high rate of diagnosis of advanced cancers, as the survival rate among advanced cancer patients is only 34.9% [3]. There is thus an urgent need for markers to help clinicians make accurate early HNSCC diagnoses, predict clinical outcomes, and provide reference for individualized medicine.

Many studies have been carried out in an effort to find predictive biomarkers to establish guidelines for the long-term prognosis of HNSCC patients. These biomarkers can be divided into two categories: 1) single molecules such as squamous cell carcinoma antigen (SCC-A), human papilloma virus (HPV), or any of the other new markers currently being studied; and 2) gene markers identified by analyzing high-throughput gene expression profiles and constructed using from several to dozens of prognostic genes. Several systems biology methods are currently being used to identify gene biomarkers related to the HNSCC prognosis and to characterize those genes [4–6]. For example, Tian et al. used weighted gene correlation network analysis and least absolute shrinkage and selection operator Cox regression to identify a 6-lncRNA signature within a gene expression profile. De et al. used gene expression meta-analysis to identify a 172-gene signature. And Zhao et al. used protein-protein interaction network analysis and Cox regression analysis to identify a 3-gene signature. All three authors tested their genetic signature using independent external data sets. However, the AUC for Zhao et al's genetic signature is not high (AUC=0.6), which means that identifying robust lncRNA signatures is still a challenge, and more investigation will be required to verify signatures. In other words, there is still an important need to identify new gene signals related to the prognosis of HNSCC through bioinformatics analysis of their biological functions.

To effectively identify a reliable gene signature associated with prognosis in HNSCC, we proposed a systematic pipeline to identify HNSCC-related gene markers. This approach enabled us to identify a 6-gene signature that can be used to effectively predict prognostic risk in HNSCC patients and provide a basis for better understanding of the molecular mechanisms underlying the prognoses of HNSCC patients.

## RESULTS

### Identification of gene sets associated with total patient survival

For The Cancer Genome Atlas (TCGA) training set samples, we used single factor regression analysis to establish the relationship between overall survival (OS) and gene expression. We identified 425 single factor Cox regression logrank P values less than 0.01. Of those, 141 genes were associated a hazard ratio (HR) > 1, and 284 with a HR < 1. The 20 genes with the highest HRs are listed in Table 1.

**Table 1:** List of the most relevant 20 genes.

**ENSG ID**	**SYMBOL**	**HR**	**coefficient**	**z score**	**P**
ENSG00000127084	*TLL2*	0.630	-0.462	-4.562	5.07E-06
ENSG00000254656	*FAM69A*	1.360	0.308	4.202	2.64E-05
ENSG00000041515	*HIST2H3PS2*	1.337	0.290	4.200	2.67E-05
ENSG00000126353	*TMCO1*	0.647	-0.435	-3.969	7.22E-05
ENSG00000115085	*MAP2K7*	0.649	-0.432	-3.916	9.01E-05
ENSG00000170482	*SPINK1*	0.538	-0.620	-3.896	9.80E-05
ENSG00000162545	*GLYCTK*	1.503	0.407	3.880	0.0001
ENSG00000125910	*ERRFI1*	0.638	-0.449	-3.838	0.0001
ENSG00000174652	*MSANTD3*	0.674	-0.395	-3.795	0.0001
ENSG00000197540	*BTLA*	0.655	-0.423	-3.722	0.0001
ENSG00000184903	*ATP6V0E1*	1.392	0.331	3.719	0.0001
ENSG00000132465	*CALML5*	0.692	-0.369	-3.698	0.0002
ENSG00000089199	*MORF4L2*	1.382	0.324	3.683	0.0002
ENSG00000189319	*ZZEF1*	0.696	-0.362	-3.674	0.0002
ENSG00000198198	*TYK2*	0.692	-0.369	-3.609	0.0003
ENSG00000153531	*EOMES*	1.318	0.276	3.553	0.0003
ENSG00000159176	*PTGR1*	1.359	0.307	3.552	0.0003
ENSG00000127241	*GPR171*	0.598	-0.515	-3.537	0.0004
ENSG00000197992	*LIMD2*	0.617	-0.483	-3.537	0.0004
ENSG00000182866	*DCP2*	0.700	-0.357	-3.494	0.0004

### Recognition of gene sets for genome variation

**Figure 1:**
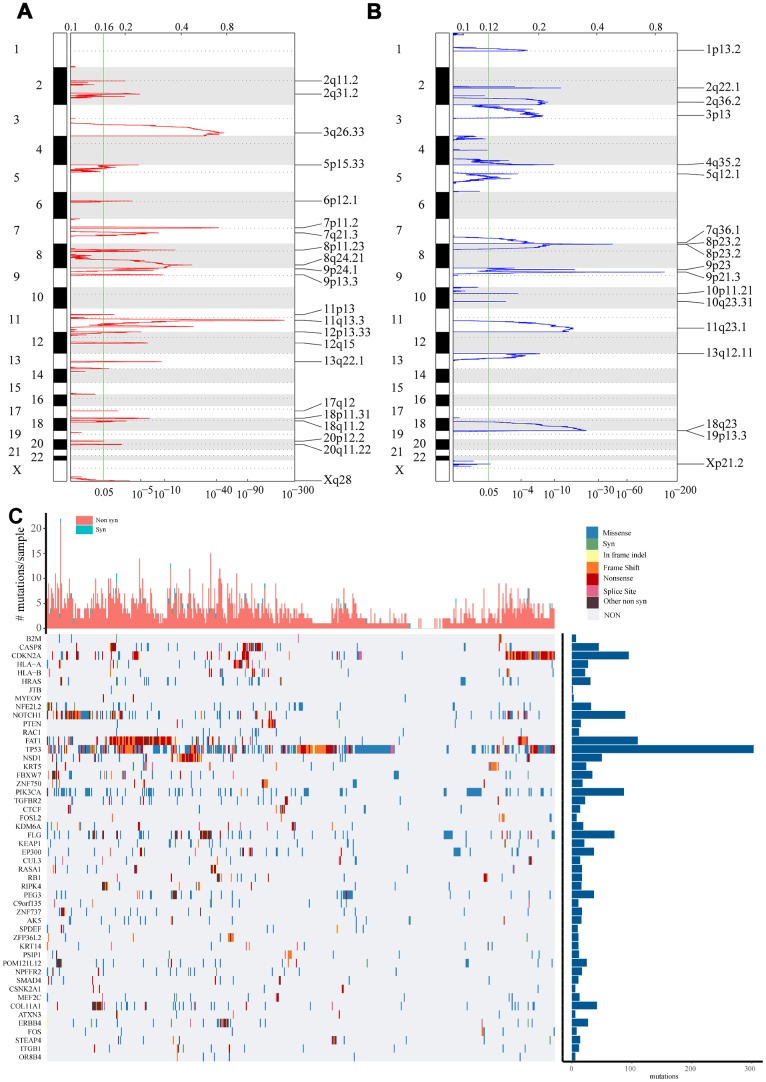
**Identification of genes with significant amplification or deletion**. (**A** and **B**) The mRNA located in the focal CNA peaks are HNSCC-related. False-discovery rates (q values) and scores from GISTIC 2.0 for alterations (x-axis) are plotted against genome positions (y-axis). Dotted lines indicate the centromeres. Amplifications (**A**) are shown in red, deletion (**B**) in blue. The green line represents 0.25 q value cut-off point that determines significance. (**C**) Top 50 genes with the most significant mutations. The bar chart above shows the total number of synonymous and non-synonymous mutations in each patient's top 50 genes. The bar chart on the right shows the number of samples in which the 50 genes were mutated in all samples. The different colors in the thermogram indicate the type of mutation; gray indicates no mutation.

Using copy number variation data in TCGA, we used GISTIC 2.0 to identify genes exhibiting significant amplification or deletion. Significantly amplified fragments within the genomes, including *EGFR* at 7p11.2 (q value = 2.28E-43), *FGF1* at 8p11.23 (q value = 3.94E-14), and *ERBB2* at 17q12 (q value = 0.0050541) (Figure 1A). In total, 247 genes were amplified (Supplementary Table 1). Genes that were significantly missing from the genome included *CDKN2A* at 9p21.3 (q value = 5.28E-149), *CDK5* at 7q36.1 (q value = 1.87E-06), and *PTEN* at 10q23.31 (q = 0.0032849) (Figure 1B). A total of 901 genes were identified as missing from the genome.

Using TCGA mutation annotation data with Mutsig2, we identified genes with significant mutations. A total of 302 genes with significant mutation frequencies were detected (Supplementary Table 2). The most significant types mutations in the Top 50 genes were synonymous mutations, missense mutations, frame insertions or deletions, frame movement, nonsense mutations, distribution of shear sites, and other non-synonymous mutations (Figure 1C). It was clear that there were differences in the mutations to different genes, including *B2M, CDKN2A, PTEN, TP53* and *FBXW7*. A common feature, however, is that mutation of these genes is reported to be closely related to the occurrence and development of tumors [7–10].

### Functional analysis of genome variant genes

**Figure 2:**
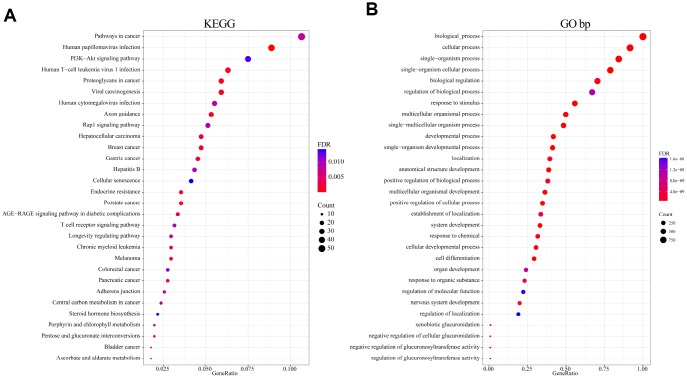
**Functional enrichment analysis of 1321 genome variant genes.** (**A**) Enriched KEGG biological pathways. (**B**) Enriched GO terms in the “biological process” category. Different colors indicate different significances, while different sizes indicate the number of genes.

To analyze the function of genomic variant genes, we integrated 1321 amplified or deleted genes and significantly mutated genes identified based on copy number variation. Gene Ontology (GO) biological process and Kyoto Encyclopedia of Genes and Genomes (KEGG) functional enrichment analyses were then performed. The results of the KEGG enrichment analysis revealed that Pathways in cancer, HPV infection, PI3K-Akt signaling pathway, Human T-cell leukemia virus 1 infection, Human cytomegalovirus infection, and numerous other related KEGG biological pathways are important to the development of cancer (Figure 2A). GO terms such as developmental process, positive regulation of cellular process, cell differentiation, and regulation of localization were mainly enriched in the “biological process” category (Figure 2B). These terms were also closely related to the occurrence and development of cancer; that is, these genes exhibiting genomic variation are closely related to cancer.

### Identification of a 6-gene signature for head and neck cancer survival

**Figure 3:**
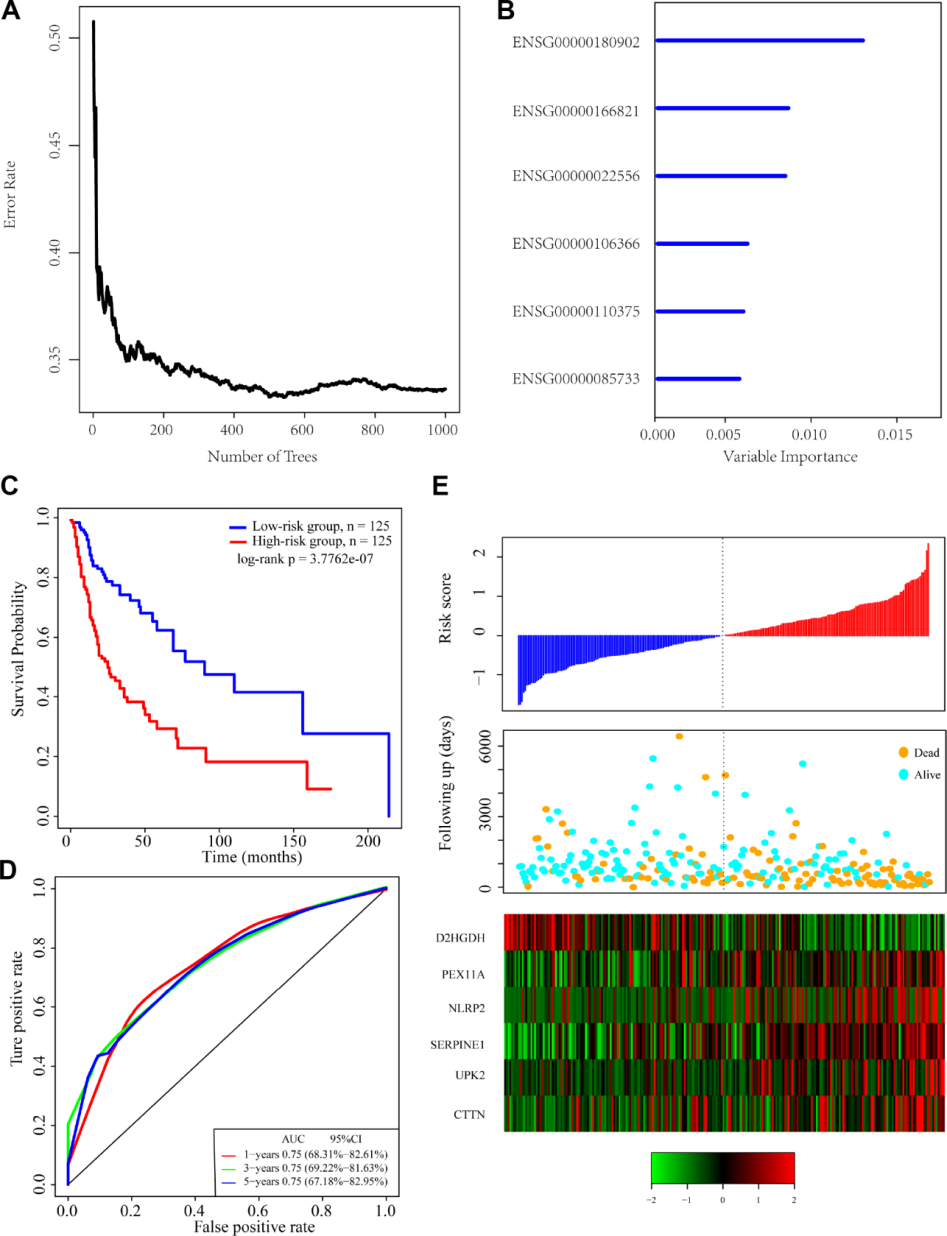
**Identification of genomic variant genes and prognosis-related genes in head and neck cancer.** (**A**) Relationship between the error rate and number of classification trees. (**B**) Importance the sequencing of 6 out-of-bag genes. (**C**) Distribution of the 6-gene signature in Kaplan-Meier survival curve for the TCGA training set. (**D**) ROC curve and AUC for the 6-gene signature classification. (**E**) TCGA training focused on risk score, survival time, survival status, and expression of the 6-gene signature.

To identify a gene signature, we integrated genomic variant and prognosis-related genes, and then selected the intersection of the groups as candidate genes, which yielded 36 genes. We then used random forests for feature selection. The relationship between error rate and number of taxonomic trees was used to reveal genes with relative importance greater than 0.4 as the final signature (Figure 3A). Ultimately, we identified 6 genes (Table 2). The important order of the out-of-bag scores for the 6 genes is displayed in Figure 3B. A 6-gene signature was established by multivariate COX regression analysis. The Equation 1 is as follows:

**Table 2:** Six genes significantly associated with OS in the training set patients.

**Ensemble Gene ID**	**Symbol**	**HR**	**Z-score**	**P**	**Importance**	**Relative importance**
ENSG00000180902	*D2HGDH*	0.77	-2.61	8.88E-03	0.012	1
ENSG00000166821	*PEX11A*	1.33	2.98	2.87E-03	0.0076	0.6359
ENSG00000022556	*NLRP2*	1.32	2.96	3.03E-03	0.0074	0.6214
ENSG00000106366	*SERPINE1*	1.43	3.33	8.47E-04	0.0052	0.4369
ENSG00000110375	*UPK2*	1.29	2.98	2.84E-03	0.005	0.4175
ENSG00000085733	*CTTN*	1.27	2.69	7.08E-03	0.0048	0.4

Risk_a_ =-0.3929517* D2HGDH+ 0.3627132 * PEX11A + 0.3038125 * NLRP2 + 0.275704 * SERP1NE1 + 0.188539* UPK2+0.1112888* CTTN (1)

The risk score of each sample was calculated, and the samples were grouped according to the median risk score (cutoff = -0.0236503). The prognoses of the high-risk and low-risk groups significantly differed (Figure 3C). The average 1-, 3-, and 5-year AUC for the 6-gene signature was 0.75 (Figure 3D). High expression of *PEX11A, NLRP2, SERPINE1, UPK2* and *CTTN* was associated with high risk, while high expression of *D2HGDH* was associated with low risk and was a protective factor.

### Verification of the robustness of the 6-gene signature model

**Figure 4:**
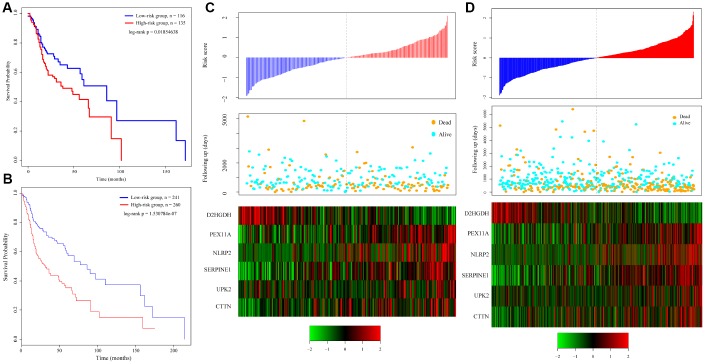
**Relation between the 6-gene signature and cancer risk.** (**A**) Kaplan-Meier curve for the test set sample. (**B**) Kaplan-Meier curve in all TCGA tumor samples. (**C**) Relationship between expression of the 6-gene signature and risk scores in test set samples. (**D**) Relationship between expression of the 6-gene signature and the risk score in all TCGA samples.

To verify the robustness of the 6-gene signature model, we first calculated a risk score for each sample in the test set. Based on the threshold for the training set, the samples were divided into two groups with significantly different prognoses (Figure 4A). The relationship between the expression of the 6 genes and the risk score was also consistent with the training set (Figure 4C). We similarly applied the model to all TCGA tumor samples and found that the low risk group fared significantly better than the high-risk group (Figure 4B). The relationship between the expression of these 6 genes and the risk score was also consistent with the training set (Figure 4D). Overall, the model effectively provided prognostic classifications with the TCGA datasets.

**Figure 5:**
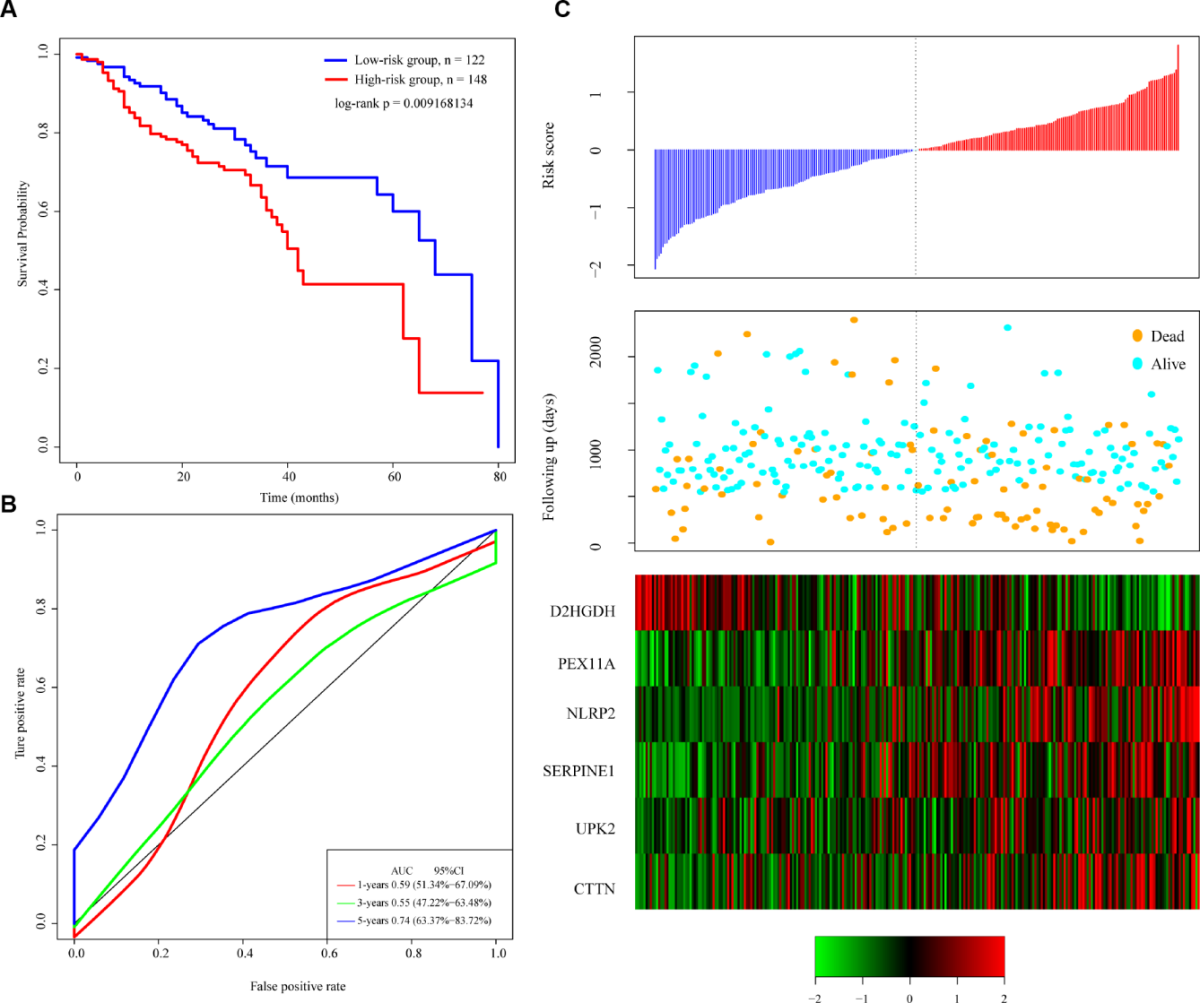
**Performance of the 6-gene signature model with GEO data.** (**A**) Kaplan-Meier survival curve distribution of 6-gene signature for the GSE65858 dataset. (**B**) ROC curve and AUC for the 6-gene signature classification. (**C**) Risk score, survival time, survival status, and expression of the 6-gene signature in the GSE65858 dataset.

To verify the classification performance of the 6-gene signature model with different data platforms, we used GEO platform data as external datasets, used the model to calculate a risk score for each sample, and used the cutoff for the training set to divide the samples into high-risk and low-risk groups. The prognosis of the low-risk group was significantly better than that of the high-risk group (Figure 5A). ROC analysis showed that the 5-year AUC was up to 0.74, compared with the training set. As in Figure 5B, the data in Figure 5C show that the relationship between the expression of the 6 genes and risk score is also consistent with the training set. Thus, the 6-gene signature model we selected was prognostic with both internal and external datasets.

### Clinical independence of the 6-gene signature model

To assess the independence of the 6-gene signature model in clinical application, we used single-factor and multi-factor COX regression to analyze HRs, 95% CIs, and P values from TCGA training set, TCGA test set, and the GSE65858 data. We systematically analyzed the clinical information from the patients as recorded in TCGA and GSE65858 datasets, including their age, sex, disease stage, pathological TNM stage, and tumor stage, as well as our 6-gene signature (Table 3). In TCGA test set, single factor COX regression analysis revealed that in the high-risk group, pathologic T3, T4, M1, and N2 all significantly correlated with survival. However, the corresponding multifactor COX regression analysis found that only high-risk group (HR = 2.17, 95% CI = 1.04-4.53, P = 0.037979), pathologic T4 (HR = 7.58, 95% CI = 1.90-30.22, P = 0.004054) and pathologic N2 (HR = 5.01, 95% CI = 1.97-12.75, P = 0.000703) had clinical independence. In TCGA test set, single factor COX regression analysis revealed that in the high-risk group, pathologic T3 and N2 correlated significantly with survival, but the corresponding multivariate COX regression analysis found that no factor had clinical independence; the HR in the high-risk group was 1.45, 95% CI = 0.67-3.12, P = 0.3389. In the GSE65858 dataset, univariate COX regression analysis revealed that the in the high-risk group, age, pathologic T3, N3, and M1 correlated significantly with survival. The corresponding multivariate COX regression analysis found that the high-risk group (HR = 1.83, 95% CI = 1.16-2.87, P = 0.0083) and age (HR = 1.03, 95% CI = 1.01-1.05, P = 0.00313) had clinical independence. These results show that our model 6-gene signature is a prognostic index independent of other clinical factors and exhibits independent predictive performance upon clinical application.

**Table 3:** Univariate and multivariate COX regression analyses of clinical factors and independence associated with prognosis.

**Variables**	**Univariate analysis**			**Multivariable analysis**		
	**HR**	**95%CI of HR**	**P**	**HR**	**95%CI of HR**	**P**
**TCGA training datasets**						
6-gene risk score						
Low risk group	1(reference)			1(reference)		
High risk group	2.66	1.79-3.92	1.060E-06	2.18	1.04-4.53	0.038
Age	1.03	1.01-1.04	8.550E-04	0.99	0.95-1.02	0.604231
Gender female	1(reference)			1(reference)		
Gender male	0.73	0.49- 1.07	0.11	0.50	0.23-1.09	0.081554
Grade 1	1(reference)			1(reference)		
Grade 2	1.78	0.95-3.32	0.07	0.91	0.25-3.18	0.88025
Grade 3 / 4	1.47	0.75-2.87	0.26	1.94	0.53-7.04	0.315185
Pathologic T 1/ T 2	1(reference)			1(reference)		
Pathologic T 3	2.26	1.28-3.95	4.53E-03	2.07	0.51-8.37	0.309
Pathologic T 4	2.46	1.50-4.00	3.19E-04	7.59	1.90-30.22	0.004
Pathologic N 0	1(reference)			1(reference)		
Pathologic N 1	0.89	0.37-2.08	0.782	0.76	0.19-3.01	0.694
Pathologic N 2	2.29	1.40-3.72	0.001	5.02	1.97-12.75	0.001
Pathologic M 0	1(reference)			1(reference)		
Pathologic M 1/ M X	1.31	0.66-2.55	0.433	1.16	0.43-3.08	0.76
Tumor stage I	1(reference)			1(reference)		
Tumor stage II	1.08	0.23-4.90	0.918	0.55	0.03-9.85	0.686335
Tumor stage III	1.40	0.30-6.42	0.667	0.77	0.05-10.60	0.845959
Tumor stage IV	2.75	0.67-11.22	0.158	0.27	0.02-3.63	0.32
**Validation cohort, TCGA test datasets and GSE65858**						
**TCGA test datasets**						
6-gene risk score						
Low risk group	1(reference)			1(reference)		
High risk group	1.62	1.08- 2.42	0.020	1.45	0.67-3.12	0.339
Age	1.01	0.98-1.02	0.444	1.00	0.96-1.03	0.993
Gender female	1(reference)			1(reference)		
Gender male	0.84	0.55-1.28	0.420	0.45	0.19-1.01	0.054
Grade 1	1(reference)			1(reference)		
Grade 2	1.86	0.92-3.77	0.08	0.90	0.28-2.88	0.865
Grade 3	1.57	0.73-3.36	0.24	1.54	0.42-5.52	0.508
Pathologic T 1/ T 2	1(reference)			1(reference)		
Pathologic T 3	1.84	1.09-3.11	0.022	1.76	0.47-6.54	0.399
Pathologic T 4	1.36	0.84-2.21	0.213	1.20	0.35-4.07	0.770
Pathologic N 0	1(reference)					
Pathologic N 1	1.14	0.57-2.25	0.706	1.74	0.50-6	0.379
Pathologic N 2	2.49	1.50-4.12	0.000	1.87	0.65-5.32	0.239
Pathologic N 3	2.90	0.87-9.6	0.082	1.86	0.26-12.82	0.529
Pathologic M 0	1(reference)			1(reference)		
Pathologic M 1	1.05	0.49-2.21	0.905	1.34	0.54-3.28	0.524
Tumor stage I	1(reference)					
Tumor stage II	2.94	0.34-0.66	0.157	0.55	0.03-9.20	0.677
Tumor stage III	3.04	0.32-0.70	0.137	1.00	0.08-11.42	0.997
Tumor stage IV	4.03	0.24-0.98	0.053	1.46	0.12-17.69	0.765
**GSE65858**						
6-gene risk score						
Low risk group	1(reference)			1(reference)		
High risk group	1.75	1.13-2.67	0.011	1.83	1.16-2.87	0.008
Age	1.03	1.00-1.04	0.01	1.03	1.01-1.05	0.00
Gender female	1(reference)			1(reference)		
Gender male	1.05	0.61-1.77	0.87	1.02	0.59-1.75	0.94
Pathologic T 1	1(reference)			1(reference)		
Pathologic T 2	0.49	0.21-1.15	0.10	0.53	0.15-1.78	0.31
Pathologic T 3	1.73	0.81-3.67	0.15	1.61	0.55-4.69	0.38
Pathologic T 4	1.89	0.91-3.87	0.08	1.22	0.42-3.53	0.71
Pathologic N 0	1(reference)			1(reference)		
Pathologic N 1	0.36	0.12-1.02	0.06	0.34	0.10-1.08	0.07
Pathologic N 2	1.60	0.99-2.56	0.05	1.03	0.51-2.05	0.94
Pathologic N 3	2.93	1.34-6.42	0.01	1.27	0.41-3.84	0.67
Pathologic M 0	1(reference)			1(reference)		
Pathologic M 1/ M X	3.71	1.63-8.4	0.00	2.18	0.70-6.79	0.18
Tumor stage I	1(reference)			1(reference)		
Tumor stage II	0.39	0.11-1.33	0.13	0.60	0.10-3.40	0.56
Tumor stage III	0.46	0.14-1.45	0.19	0.66	0.13-3.31	0.61
Tumor stage IV	1.49	0.60-3.70	0.39	1.14	0.24-5.20	0.87

### GSEA analysis of pathway differences enriched in the high-risk and low-risk groups

**Figure 6:**
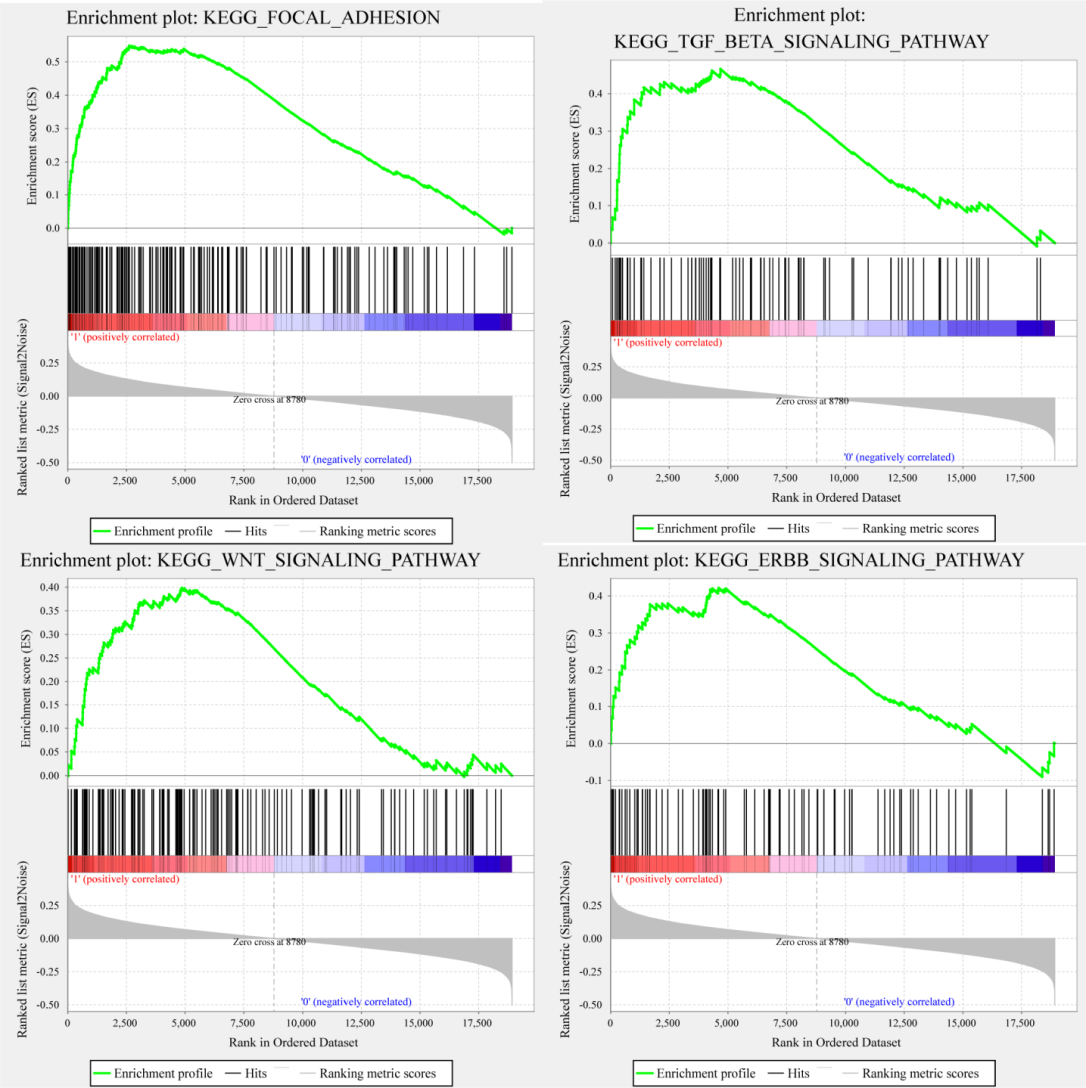
**GSEA showing four pathways enriched in the high-risk group.** GSEA enrichment results for focal adhesion, TGF-β signaling pathway, WNT signaling pathway, and ERBB signaling pathway.

GSEA was used in TCGA training to analyze the pathways significantly enriched in the high-risk and low-risk groups. Twenty enriched pathways were detected (Supplementary Table 3), including focal adhesion, TGF-β signaling pathway, WNT signaling pathway, and ERBB signaling pathway, all of which are closely related to tumor occurrence, development and metastasis. Notably, these pathways are significantly enriched in the high-risk samples (Figure 6).

## DISCUSSION

In terms of prognosis, head and neck cancer is a highly heterogeneous disease in that survival times vary substantially among patients with similar TNM stages. With the diagnosis and treatment of head and neck cancers at earlier stages, traditional clinicopathological indicators such as tumor size, vascular invasion, portal vein thrombus and TNM stage have proven inadequate for predicting individual outcomes, especially risk stratification, as no one-size-fits-all treatment strategy appears to be effective [11, 12]. Consequently, screening prognostic molecular markers that adequately reflect the biological characteristics of tumors would be of great significance for individualized prevention and treatment of head and neck cancer patients. In the present study, we analyzed the expression profiles of 771 head and neck cancer samples from TCGA and the GEO and identified 6 genes robustly associated with OS. This signature is independent of other clinical factors.

Gene signatures are currently being used in clinical practice. Two examples are Oncotype DX [13–15], which provides a breast cancer recurrence score based on expression of 21 genes, and Coloprint, which provides a colon cancer recurrence score based on expression of 18 genes [16–18]. Results obtained with these assays have shown that screening new prognostic cancer markers based on gene expression profiles is a promising high-throughput molecular identification method. In that regard, Tian et al. [6] identified a 6-gene signature, but the verification set AUC was only about 0.65, and Zhao et al. [4] identified a 3-gene signature, but the AUC was only about 0.6. In addition, De et al. 5] identified a 172-gene signature through meta-analysis of gene expression. Although the AUC is high, the large number of genes that needs to be detected makes this analysis impractical for clinical use. By contrast, our 6-gene signature has a high AUC using only 6 genes, which makes it conducive to clinical application.

The six genes in our signature include *PEX11A, NLRP2, SERPINE1, UPK*, and *CTTN* as risk factors, and *D2HGDH* as a protective factor. It has been reported that *NLRP2* can be used as a marker of leukemia [19] and has an important impact on the prognosis after stem cell transplantation [20]. *SERPINE1* is closely related to prognosis in ovarian cancer, gastric cancer, thyroid cancer and other tumors [21–25]. *CTTN* is closely related to prognosis in head and neck cancer, esophageal cancer, thyroid cancer, and glioma, among others [26–29]. *D2HGDH* is a marker of colorectal cancer, glioma, and prostate cancer [30–33]. *PEX11A* and *UPK* have not been previously reported to be related to cancer. Ours is the first study to suggest that they can be used as new prognostic markers of head and neck cancer. At the same time, our GSEA results show that the 6-gene signature enrichment significantly correlates with pathways and biological processes associated with the occurrence and development of HNSCC. This indicates that our model has potential clinical application value and could provide a potential target for diagnosis and for development of new targeted therapies that include, for example, novel alkylating agents [34, 35].

Although we have identified potential candidate genes affecting tumor prognosis using bioinformatics technology with large samples, our study has limitations. First, the sample lacks some clinical follow-up information, so we did not consider factors such as the presence of other health conditions to differentiate prognostic biomarkers. Second, the results obtained using bioinformatics analysis alone are insufficient and need to be confirmed through experimental verification. Therefore, further genetic and experimental studies with larger samples and experimental validation are needed.

## CONCLUSIONS

In our study, we developed a 6-gene signature prognostic stratification system, which has good AUC values in both the training set and validation set, and is independent of other clinical features. Compared to clinical features, gene classifiers can improve survival risk prediction. We therefore recommend using this classifier as a molecular diagnostic test to assess prognostic risk in patients with head and neck cancer.

## MATERIALS AND METHODS

### Data acquisition and processing

The FPKM data of TCGA RNA-Seq downloaded from the UCSC Cancer Browser (https://xenabrowser.net/datapages/) contained 546 samples. The clinical follow-up information contained 612 samples, and the copy number variation data on the SNP 6.0 chip contained 519 samples. The mutation annotation information (MAF) downloaded from the GDC client contained 504 samples. The standardized tables of the GSE658587 [36] data set were downloaded from the GEO. For TCGA RNAseq data, 501 tumor samples with follow-up information were chosen and randomly divided into two groups: a training set (N = 250) (Supplementary Tables 4 and 5) and a test set (N = 251) (Supplementary Tables 6 and 7). The GSE65858 data set was used as an external verification set for each group of sample information (Table 4).

**Table 4:** Clinical information statistics for three datasets.

**Characteristic**		**TCGA training datasets (n=250)**	**TCGA test datasets (n=251)**	**GSE65858 (n=270)**
**Age(years)**	<=50	38	50	41
	>50	212	201	229
**Survival Status**	Living	136	146	176
	Dead	141	105	94
**Gender**	female	68	66	47
	male	182	185	223
**Grade**	G 1	34	28	
	G 2	136	163	
	G 3	68	51	
	G 4	2	0	
**Pathologic_T**	T 1	17	28	35
	T 2	67	65	80
	T 3	48	48	58
	T 4	89	63	97
**Pathologic_N**	N 0	80	90	94
	N 1	26	39	32
	N 2	94	72	132
	N 3	2	5	12
**Pathologic_M**	M 0	94	93	263
	M 1/ M X	32	30	7
**Tumor Stage**	Stage I	9	16	18
	Stage II	37	32	37
	Stage III	30	48	37
	Stage IV	141	120	178

### Univariate Cox proportional risk regression analysis

As described previously by Jin-Cheng et al. [37], univariate Cox proportional hazard regression analysis was performed with each immune gene to screen out genes significantly associated with OS in the training data set. P < 0.01 was chosen as the threshold.

### Analysis of copy number variation data

GISTIC software is widely used to detect both broad and focal (potentially overlapping) recurring events. We used GISTIC 2.0 [38] to identify genes exhibiting significant amplification or deletion. The parameter threshold was that the length of the amplification or deletion was more than 0.1, and the fragment was P < 0.05.

### Gene mutation analysis

We used Mutsig 2.0 software with TCGA mutation data to identify genes with significant mutations in their MAF files. The threshold used was P < 0.05.

### Construction of a prognostic gene signature

We chose the genes that were significantly related to OS and genes that were exhibited amplification, deletion or mutation. We further used the Random Survival Forest algorithm to rank the importance of prognostic genes. Like Jin et al. [39], we used the R package random Survival Forest to screen the prognostic genes. We set the number of Monte Carlo iterations to 100 and the number of steps forward to 5, and identified the genes whose relative importance as characteristic genes was greater than 0.4. In addition, we carried out a multivariate Cox regression analysis, and the following risk scoring model was constructed using the Equation 2:

$$RickScore\text{​}\;=\sum_{k-l}^{n}Exp_{k}*e^{HR}{}_{k}$$ (2)

where N is the number of prognostic genes, $Exp_{k}$ is the expression value of the prognostic genes, and $e^{HR}{}_{k}$ is the estimated regression coefficient of genes in the multivariate Cox regression analysis.

### Functional enrichment analyses

GO and KEGG pathway enrichment analysis was performed using the R package cluster profiler for genes [40], to identify over-represented GO terms in three categories (biological processes, molecular function and cellular component) as well as over-represented KEGG pathway terms. For this analyses, a false discovery rate < 0.05 was considered to indicate statistical significance.

GSEA [41] was performed using the http://software.broadinstitute.org/gsea/downloads.jsp website with the MSigDB [42] C2 Canonical pathways gene set collection, which contains 1320 gene sets. Gene sets with a false discovery rate < 0.05 after performing 1000 permutations were considered to be significantly enriched.

### Statistical analysis

When the median risk score in each data set was used as a cutoff to compare survival risk between high-risk and low-risk groups, a Kaplan-Meier (KM) curve was drawn. Multivariate Cox regression analysis was used to test whether gene markers were independent prognostic factors. Significance was defined as P < 0.05. All analyses were performed using R 3.4.3.

## Supplementary Material

Supplementary Table

Supplementary Table 1

Supplementary Table 2

Supplementary Table 3

Supplementary Table 4

Supplementary Table 5

Supplementary Table 6
