# Supplementary material for: Six-gene signature for predicting survival in patients with head and neck squamous cell carcinoma
Source: Aging (Albany NY). 2020 Jan 12;12(1):767–83. doi: 10.18632/aging.102655 (PMC6977678; doi:10.18632/aging.102655)
Supplement: Supplementary Table [file aging-12-102655-s005..pdf]

SUPPLEMENTARY TABLES

Please browse Full Text version to see the data of Supplementary Tables 1–6

Supplementary Table 1. Amplified 247 genes.

Supplementary Table 2. 302 genes with significant mutation frequencies.

Supplementary Table 3. Information of training set.

Supplementary Table 4. Clinical information of training set.

Supplementary Table 5. Information of test set.

Supplementary Table 6. Clinical information of test set.

Supplementary Table 7. The top twenty enriched pathways.

NAME	SIZE	ES	NES	NOM p-val	FDR q-val	FWER p-val
KEGG_ADHERENS_JUNCTION	73	0.604583	1.982844	0	0.076978	0.042
KEGG_FOCAL_ADHESION	199	0.548716	1.832598	0.014199	0.257997	0.24
KEGG_PATHOGENIC_ESCHERICHIA_COLI_INFECTION	55	0.478323	1.751252	0.00789	0.378554	0.417
KEGG_RENAL_CELL_CARCINOMA	70	0.464708	1.714178	0.00996	0.388839	0.51
KEGG_REGULATION_OF_ACTIN_CYTOSKELETON	212	0.423736	1.680424	0.010267	0.402515	0.592
KEGG_ECM_RECEPTOR_INTERACTION	83	0.595027	1.672061	0.061181	0.35479	0.61
KEGG_AXON_GUIDANCE	128	0.43319	1.669205	0.006424	0.310781	0.615
KEGG_TGF_BETA_SIGNALING_PATHWAY	85	0.465713	1.622249	0.027668	0.380817	0.719
KEGG_WNT_SIGNALING_PATHWAY	149	0.398066	1.59946	0.010616	0.392898	0.76
KEGG_GAP_JUNCTION	89	0.424439	1.583867	0.023207	0.388653	0.785
KEGG_THYROID_CANCER	29	0.468404	1.56782	0.044834	0.391882	0.81
KEGG_ERBB_SIGNALING_PATHWAY	87	0.420793	1.547303	0.025263	0.403438	0.829
KEGG_ARRHYTHMOGENIC_RIGHT_VENTRICULAR_ CARDIOMYOPATHY_ARVC	74	0.473225	1.542216	0.066528	0.383884	0.835
KEGG_TIGHT_JUNCTION	130	0.381005	1.530401	0.041152	0.382432	0.847
KEGG_PATHWAYS_IN_CANCER	324	0.360142	1.497865	0.035124	0.433172	0.884
KEGG_VIBRIO_CHOLERAE_INFECTION	52	0.40149	1.483408	0.036961	0.442476	0.894
KEGG_PANCREATIC_CANCER	70	0.408252	1.476431	0.065606	0.43181	0.897
KEGG_ALPHA_LINOLENIC_ACID_METABOLISM	19	-0.72946	-2.11189	0	0.012797	0.009
KEGG_LINOLEIC_ACID_METABOLISM	29	-0.64152	-1.84077	0.001976	0.215058	0.23
KEGG_ARACHIDONIC_ACID_METABOLISM	57	-0.52228	-1.82148	0.005952	0.168108	0.266
KEGG_INTESTINAL_IMMUNE_NETWORK_FOR_IGA_ PRODUCTION	46	-0.61053	-1.62009	0.067864	0.626243	0.702
